# A single-nucleotide polymorphism (SNP) multiplex system: the association of five SNPs with human eye and hair color in the Slovenian population and comparison using a Bayesian network and logistic regression model

**DOI:** 10.3325/cmj.2012.53.401

**Published:** 2012-10

**Authors:** Vanja Kastelic, Katja Drobnič

**Affiliations:** 1 National Forensic Laboratory, General Police Directorate, Police, Ministry of the Interior, Ljubljana, Slovenia; 2 Faculty of Criminal Justice and Security, University of Maribor, Ljubljana, Slovenia

## Abstract

**Aim:**

To analyze two phenotype characteristics – eye and hair color – using single-nucleotide polymorphisms (SNPs) and evaluate their prediction accuracy in Slovenian population.

**Methods:**

Twelve SNPs (*OCA2* – rs1667394, rs7170989, rs1800407, rs7495174; *HERC2 –* rs1129038, rs12913832; *MC1R* – rs1805005, rs1805008; *TYR* – rs1393350; *SLC45A2* – rs16891982, rs26722; *SLC24A5* – rs1426654) were used for the development of a single multiplex assay. The single multiplex assay was based on SNaPshot chemistry and capillary electrophoresis. In order to evaluate the accuracy of the prediction of eye and hair color, we used the logistic regression model and the Bayesian network model, and compared the parameters of both.

**Results:**

The new single multiplex assay displayed high levels of genotyping sensitivity with complete profiles generated from as little as 62 pg of DNA. Based on a prior evaluation of all SNPs in a single multiplex, we focused on the five most statistically significant in our population in order to investigate the predictive value. The two prediction models performed reliably without prior ancestry information, and revealed very good accuracy for both eye and hair color. Both models determined the highest predictive value for rs12913832 (*P* < 0.0001), while the other four SNPs (rs1393350, rs1800407, rs1805008, and rs7495174) showed additional association for color prediction.

**Conclusion:**

We developed a sensitive and reliable single multiplex genotyping assay. More samples from different populations should be analyzed before this assay could be used as one of the supplemental tools in tracing unknown individuals in more complicated crime investigations.

Height, face structure, pigmentation of the eye, hair, and skin, the presence of freckles, and male baldness make up human externally visible characteristics (EVC). To be able to predict eye and hair color based solely on biological material left behind at a crime scene or obtained from dismembered missing persons, or even of disaster victims, is one of the major expectations from the routine forensic work in the near future (1). However, genetic understanding of human appearance is still in its infancy, mainly due to the fact that all EVCs are polygenic traits. This means that yields from a large number of different genes and the expression of these genes are further influenced by mutual interactions and environmental interactions (2). Above all, molecular mechanisms and functional protein assays must also be considered in order to really understand how allelic variation in pigmentation genes could result in such a diversity of phenotypes in different human populations (3). The human eye (iris) and hair color are one of the most highly polymorphic phenotypes in people of European origin. The non-brown iris colors and red hair are generally features of European origin resulting from positive selection in early European history. There are several hypotheses for positive selection that mainly occurred in the Baltic region and Northern Europe. These are most likely: UV exposure causing skin cancer, vitamin D deficiency, and even sexual selection (4,5). Most EVCs are complex traits with many genes and single nucleotide polymorphism (SNP) variations, so the right combination of SNPs is crucial for the correct prediction of eye and hair color. Several genome-wide association studies (GWAS) for pigmentation have revealed that SNPs within the *HERC2, OCA2, MC1R, SLC24A5, SLC45A2, TYR,* and *ASIP* (4,6-16) genes were most strongly associated with eye and hair color in European populations. The latest data have shown that the main iris color variation is associated with a highly evolutionarily preserved region in the *HERC2* gene or within the short sequence between the *HERC2* and *OCA2* genes. It is assumed that these regions represent a regulatory region controlling the constitutive expression of *OCA2* (4,11,12). As for iris color, it has also been explained that red hair color is mainly associated with polymorphisms in the *MC1R* gene (13,17). On the other hand, the variations of genes such as *SLC24A5, SLC45A, HERC2,* and *ASIP* seem to be responsible for influencing the shades of hair color from blond to black (18,19).

In order to correctly predict human eye and hair color from genetic data for the Slovenian population, we compared two alternative prediction models that are nowadays used most often in this field of forensics – the Bayesian network model and the logistic regression model. These models were developed and compared on the basis of the informative SNPs selected from our single multiplex assay.

## Material and methods

### Sample collection

The research included 105 unrelated Slovenian volunteers, 70 male and 35 female donors. Buccal swabs of the adult volunteers were collected in 2008 using a SAFE^®^ Box kit (ForensiX, Prionics AG, Zurich, Switzerland).

The eye and hair color of each volunteer were defined according to descriptions provided by the volunteers and through our observer grading. In the small number of elderly volunteers and volunteers with dyed hair, we used self-assessment for establishing natural hair color phenotypes. We then categorized each volunteer's eye and hair color into three groups. Eye color was defined as blue (44.7%), intermediate (25.7%), and brown (29.6%). For classification into blue and brown eye color, the eye must have been clearly composed of one color, regardless of its intensity. However, the classification into intermediate eye color included all other individuals with green or hazel eye colors, or those with two or more pigments within the iris (peripapillary rings or different colored spots). The hair color was defined as blond (5.7%), dark blond/light brown (41.0%), and dark brown/black (52.4%).

Red hair color was not included because our research group included only one red-haired person due to the small number of red-haired persons in the Slovenian population. A demonstration of prediction accuracy for red hair color based on only one person’s data are not statistically acceptable, so we excluded this person and limited the study to the three hair color groups. The study was approved by the National Medical Ethics Committee in Slovenia. Before donating a sample, all the volunteers signed written consents for the use of their DNA solely for scientific research.

### DNA extraction and quantification

DNA was extracted from all the samples using Chelex extraction (20). Isolated DNA was quantified using the real-time PCR method – ABI Prism 7500 Sequence Detection System (Applied Biosystems Inc., Foster City, CA, USA). The extraction yield ranged between 0.7 and 20 ng/µL per person. This broad range can be explained by the unequal shedding of buccal cells among people.

### SNP selection and multiplex design

Twelve of the SNPs (4,6-8,21) (Supplementary Table 1(web extra material 1)) that were associated with human eye and hair color in previous studies were analyzed in one multiplex assay. The PCR primers reported (Supplementary Table 2(web extra material 2)) were combined in one multiplex reaction and, as a result, the primers’ melting temperatures were verified and the potential interactions were preliminarily checked using the AutoDimer software program (22). The length of PCR fragments was limited to between 104 and 238 bp in order to meet future forensic investigations due to degraded DNA. For the single multiplex PCR, a genomic DNA extract from each individual was amplified ranging from 0.03125 and 1.0 ng in 25-µL reactions containing 1 × AmpFlSTR PCR reaction mix (Applied Biosystems), 8 mM MgCl_2_, 0.1-1.7 µM of each primer, and 1-5U AmpliTaq Gold DNA polymerase (Applied Biosystems). The following cycle program was used: denaturation at 95°C for 10 minutes followed by 35 cycles of 95°C for 30 seconds, 60°C for 30 seconds, and 72°C for 30 seconds, followed by final incubation for 70 minutes at 72°C. PCRs were performed in a Perkin-Elmer 9600 thermal cycler (Applied Biosystems).

**Table 1:** Parameters describing predictive accuracy of two developed eye color prediction models, divided into two variants for 24 Slovenian volunteers*

	Multinomial logistic regression/Bayesian network			Binary logistic regression/Bayesian network	
	blue	intermediate	brown	light	dark
AUC	1.000/1.000	0.747/0.632	0.832/0.685	0.985/0.924	0.985/0.924
Sensitivity (%)	100.0/100.0	100.0/x	71.0/14.0	88.0/86.0	100.0/94.0
Specificity (%)	100.0/100.0	63.0/100.0	90.0/100.0	88.0/100.0	x/100.0
PPV (%)	100.0/100.0	25.0/x	71.0/100.0	100.0/100.0	100.0/100.0
NPV (%)	100.0/100.0	100.0/50.0	90.0/54.0	88.0/86.0	x/94.0

*Abbreviations: AUC – area under the receiver operating characteristic (ROC) curves; PPV – positive predictive value; NPV – negative predictive value; x – zero denominator.

**Table 2:** Parameters describing predictive accuracy of two developed hair color prediction models, divided into two variants, for 24 Slovenian volunteers *

	Multinomial logistic regression/Bayesian network			Binary logistic regression/Bayesian network	
	blond	dark blond/light brown	dark brown/black	light	dark
AUC	0.913/0.714	0.723/0.445	0.832/0.543	0.929/0.878	0.929/0.878
Sensitivity (%)	x/x	50.0/x	88.0/79.0	14.0/71.0	100.0/88.0
Specificity (%)	x/100.0	80.0/88.0	95.0/100.0	100.0/100.0	100.0/100.0
PPV (%)	x/x	33.0/x	88.0/100.0	100.0/100.0	100.0/100.0
NPV (%)	x/100.0	89.0/50.0	95.0/89.0	14.0/67.0	100.0/88.0

*Abbreviations: AUC **–** area under the receiver operating characteristic (ROC) curves; PPV **–** positive predictive value; NPV **–** negative predictive value; x **–** zero denominator.

After PCR amplification, the excess PCR primers and ddNTPs were removed by addition of freshly prepared mix of shrimp alkaline phosphatase (1U/µL) (ABGene, Epsom, UK) and Exonuclease I (20U/µL) (ABGene) to 5 µL PCR products and incubation at 37°C for 1 hour followed by incubation at 75°C for 15 minutes.

Ten single base extension (SBE) primers were collected from previous reports and two were self-designed (Supplementary Table 1(web extra material 1)). To ensure varying lengths of SBE primer in order to adjust their mobility in capillary electrophoresis, GACT-tails were added to the 5′ ends of each SBE primer. A multiplex SBE reaction was performed, using an ABI Prism SNaPshot kit (Applied Biosystems) in a total reaction volume of 8 µL, containing 1 μL purified PCR products, 4 μL SnaPsho reaction mix (Applied Biosystems), and 0.01-1.7 μM of each of SBE primers and Milli-Q water. Thermal cycling for SBE was performed in a thermal cycler (Applied Biosystems), using a program for 30 cycles at 96°C for 10 seconds, at 50°C for 5 seconds, and at 60°C for 30 seconds. Excess fluorescently labeled ddNTPs were removed by adding shrimp alkaline phosphatase (1U/µL) (ABGene) and incubating them at 37°C for 45 minutes, followed by 15 minutes incubation at 75°C.

SBE products were then analyzed on an ABI Prism 3130 Genetic Analyzer (Applied Biosystems) following standard protocol (23), with a 36-cm capillary array, POP-4 polymer (Applied Biosystems), and a 5-second injection at 1.5 kV. Allele calling was performed using GeneMapperID ver. 3.2 software (Applied Biosystems) and a bin set, according to our SBE product size, was designed for our multiplex to allow automation of genotyping. To test the overall sensitivity of the multiplex assay, all the samples were analyzed in duplicate; if the results were inconsistent, additional amplifications were made.

### Analysis of approved allele calls

For each SNP locus, we determined the average peak height ratio for heterozygotes and homozygotes, and the standard deviation of the peak height was also provided. From some of the randomly selected electropherograms, the peak height of the highest background peak in each dye window was collected and an arbitrary maximum background level determined (blue and green dye – 110 RFUs, yellow dye – 80 RFUs, and red dye – 70 RFUs (relative fluorescent units). The background levels were used to calculate an arbitrary signal/noise ratio for homozygote allele calls. For heterozygote allele calls, the peak height ratio was calculated by dividing the peak height of the lower molecular weight allele by the peak height of the higher molecular weight allele for all SNPs. The right allele calls were determined for all 105 genotypes of twelve SNPs.

### Reproducibility and sensitivity

Validation of the single multiplex was conducted on samples of two individuals. Multiplex PCR performance was assessed by analyses of dilution series of genomic DNA (0.03; 0.06; 0.12; 0.25; 0.50 1.00, and 2.00 ng), amplifying with 1-5 U AmpliTaq Gold DNA polymerase (Applied Biosystems) in the reaction mix. To ensure the consistency of genotyping, two of the samples with all dilution series were analyzed in duplicates and additionally the homozygote and heterozygote peak height ranges were noted for each locus.

### Statistical analysis

In order to evaluate allele frequencies, locus-by-locus molecular variance (AMOVA), the Hardy-Weinberg equilibrium (HWE), and linkage disequilibrium (LD), we used Arlequin, version 3.1 (24). The *P* values were corrected using the Bonferroni correction for multiple testing (*P* > 0.0045) for the final determination of SNPs in linkage disequilibrium.

The statistical analysis for the prediction of phenotypes from genotypes was based on defining eye color as blue, intermediate, and brown, and hair color as blond, dark blond/light brown, and dark brown/black in the first step. Additionally we simplified the classification for both eye and hair color with only two stages: light and dark. For statistically relevant SNPs the probability values for phenotype prediction were first calculated based on multinomial/binary logistic regression (17,25) using SPSS 19.0 for Windows (SPSS Inc., Chicago, IL, USA) and second based on likelihood ratios (LRs) liable to the Bayesian approach using the excel macro of the Branicki group (26). In order to evaluate the predictive accuracy of both models, we randomly split our samples into a model-building set consisting of 80 individuals (77%) and a model verification set comprising the remaining 24 (33%) individuals. When working with the Bayesian network model, the *a priori* conditional probabilities of colors were entered as having the same value (0.33 – three color description, 0.5 – two color description), because we rarely have prior knowledge on the ancestry of the identified subject. For worldwide distribution, a threshold of 0.7 predicted eye or hair color probability was used for categorization. When the probability values were under this fixed threshold, the eye or hair color was predicted as undefined. This cut-off was based on the receiver operating characteristics (ROC) curve, or area under the receiver operating characteristic curve (AUC), derived from previous studies. AUC is the integral of ROC curves, which ranges from 0.5, representing a total lack of prediction, to 1.0, representing perfect prediction (25).

## Results

### Multiplex design and protocol

The single multiplex assay was designed based on twelve PCR fragments of lengths less than 240 bp, and was therefore useful for casework DNA samples. Ten previously reported SBE primers and two newly designed primers were evenly separated by 5 bp in the region of 30-65 bp in length for precise marker differentiation. Allele peaks were only called if they were above 50 RFU in their respective size range within a custom-designed bin set for our single multiplex, using GeneMapperID, version 3.2 software.

**Figure 1:**
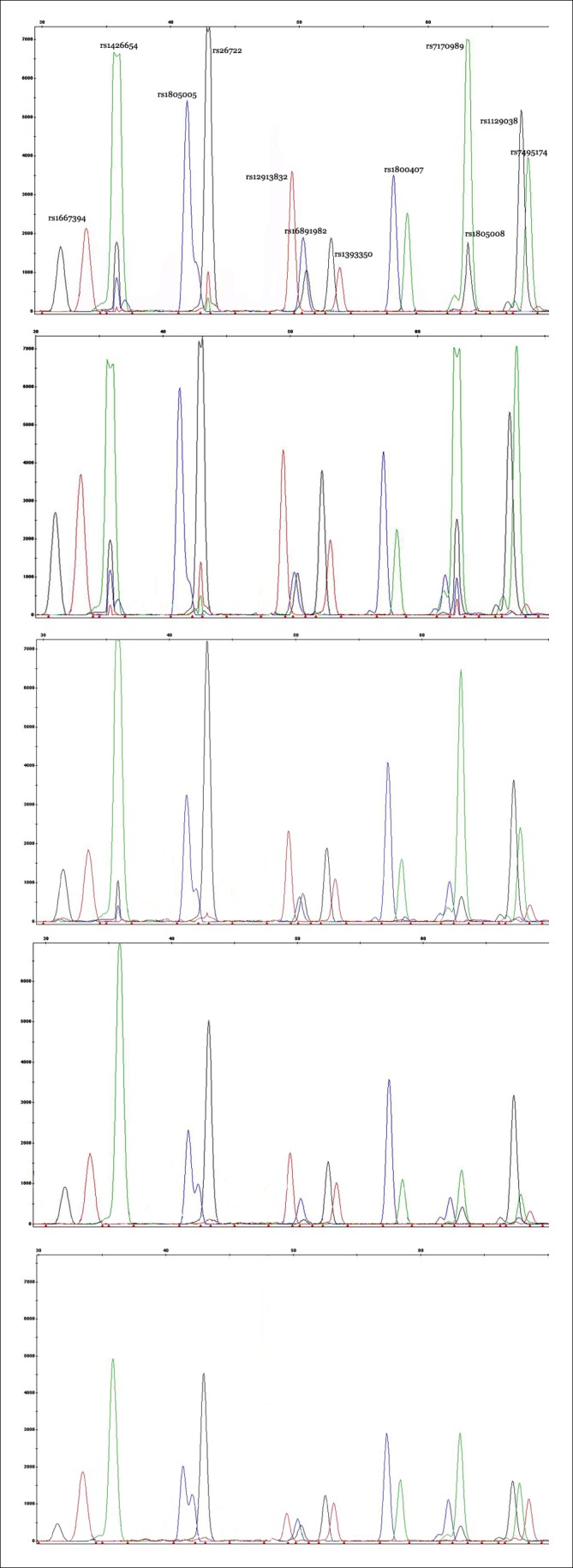
Sensitivity testing of our multiplex assay. Multiplex single base extension (SBE) products for 1.0; 0.5; 0.25; 0.125, and 0.0652 ng of DNA input for one random sample.

This single multiplex assay worked optimally at 1 ng of template DNA using 1U of the AmpliTaq Gold DNA polymerase for PCR amplification. The sensitivity of the multiplex assay rised remarkably by increasing the concentration of the polymerase up to 5U. Using these amounts of polymerase for PCR amplification, the drop-out partially appeared at only 62 pg of DNA input or even less (Figure 1). For our assay, the homozygote and heterozygote peak heights for all SNPs were balanced inter-loci. Still, we could not achieve an inter-loci balance for all SNPs, which did not in any way affect the genotyping accuracy.

The homozygote and heterozygote average peak heights of each locus from all 105 samples of 1 ng of DNA input were calculated and the relations were from 1500 to 5500 RFU and from 500 to 3000 RFU, respectively. The heterozygote ratio for SNPs rs26722 and of rs16891982 could not be determined precisely and, as a result of their rarity in the European population, sufficient data could not be collected. In order to understand a more balanced profile for SNP rs16891982, we increased this final concentration from 0.25 µM up to 0.65 µM of the same SBE primer, and the results in the changed multiplex assay were the same as had been shown by the monoplex genotyping assay for the aforementioned SNP for all 105 samples.

### Population analysis

In order to determine how well the Slovenian sample population reflected its ancestry, the allele frequencies for the twelve SNPs selected were compared with the data from the International HapMap consortium (27). The International HapMap project is a multi-country effort to identify and catalog genetic variants. For the evaluation of the Slovenian population we compared it with HapMap data of Utah residents with European ancestry (CEU). The allele frequency of our population was very similar to that of the HapMap data. A major difference was noticed for SNP rs16891982, assuming that this was for the same reason stated above – its rarity of heterozygote SNP profiles, and for this reason sufficient data could not be collected. The SNP rs1426654 was the only one that was monomorphic (homozygote for the allele A/A) and showing the same data as presented on the HapMap project (Supplementary Table 3(web extra material 3)). This indicated that rs1426654, derived threonine allele, was fixed in the European population.

We analyzed the fact that all 105 individuals were homozygotes for the derived allele A/A. Based on that, this marker was not included further in calculating the prediction accuracy for pigmentation color. From the 105 Slovenian samples we eliminated only one person, this being the only person with red hair in our population (0.9% of the Slovenian population). This low frequency of red hair color was expected for the general Slovenian population and additionally confirmed that red haired individuals were more prevalent in the Baltic region and Northern Europe populations (28).

The linkage disequilibrium (LD) between all the SNPs was calculated. As had been previously indicated (12), we established that SNPs rs1129038 and rs12913832 were in perfect linkage disequilibrium and could be considered a single haplotype owing to their close position on the chromosome 15. SNP allele haplotypes were significantly associated with eye and hair color, but due to linkage disequilibrium we decided to use only rs12913832 for further prediction information. We could also conclude that two SNPs, rs1667394 and rs7170989, were in linkage disequilibrium with SNP rs1800407 for the same reason as previously stated, and we also included only SNP rs1800407 in the statistical models. Finally, after using the Bonferroni correction for multiple testing, we did not detect any significant departures from the Hardy-Weinberg equilibrium (*P* values ranged from 0.1889 to 1.0000) for all the 11 analyzed SNPs (SNP rs1426654 was monomorphic).

### Eye and hair color prediction models

In order to evaluate the predictive accuracy for eye and hair color, we established the SNP position that could be statistically significant in our study to determine the accurate prediction. In the prediction model, we did not include the markers that were monomorphic (rs1426654) or in linkage disequilibrium with other SNPs (rs1129038, rs1667394, and rs7170989). With the elimination of these four SNPs, we first used the eight remaining SNPs (rs1800407, rs7495174, rs12913832, rs1805008, rs1805005, rs1393350, rs26722, rs16891982) to simultaneously test for the effect on a dichotomous dependent variable with binary logistic regression (where eye color was classified as blue vs non-blue, intermediate vs non-intermediate, and brown vs non-brown; and hair color was classified as blond vs non-blond, dark blond/light brown vs non-dark blond/light brown, and dark brown/black vs non-dark brown/black). Based on the results we selected the five SNPs (rs1800407, rs7495174, rs12913832, rs1805008, rs1393350) that were statistically significant (*P* < 0.05) for the group for which the analysis was made. The multinomial/binary logistic regression model and the Bayesian network model (25,26) were developed and tested for eye and hair color accuracy prediction. The first variant for both models assumed prediction of pigment color being divided into three groups: blue, intermediate, brown (eye color); and blond, dark blond/light brown, and dark brown/black (hair color). The second variants for both models were reduced to only two states – light and dark for eye and hair color.

For blue eye color using both statistical models for 24 samples, the prediction was completely accurate, when using three categorized eye colors. When using the logistic model for evaluation, high values were also obtained for brown eye color, with AUC of 0.832, but were least accurate for intermediate eye color, with AUC of 0.747. For blue eye color categorization, we got a 100% correct call rate (sensitivity), which means that all blue-eyed individuals were predicted correctly (24/24). The sensitivity was also high for intermediate (100%) eye color and relatively high for brown eye color (71%) (Table 1). The highest specificity was obtained for blue eye color (100%), which means that among all non-blue eye colored individuals, all of them were recognized correctly. High specificity was also obtained for brown (90%) eye color and much lower for intermediate (63%) eye color.

The AUC for eye color defined as light vs dark was calculated for both colors to equal 0.985. The sensitivity in this categorization of eye color was also high and even reached 100% for dark eye color and 88% for light eye color. All the described results were mostly summarized only for the multinomial logistic regression model. However, most values based on the Bayesian model variant were slightly lower, but still in the same range (Table 1).

The highest value for AUC, 0.913, was obtained for blond hair color and even for dark brown/black hair color, 0.832, when using the logistic model for hair color categorized into three groups. As indicated for eye color prediction, for hair color the AUC values were also slightly higher when using the logistic model in comparison to the Bayesian network model. Sensitivity (88%) and also specificity (95%) were highest for dark brown/black hair color and they could not be extended for the other two hair color groups, presumably due to their small size sample in the model verification set of 24 Slovenian volunteers.

The AUC for hair color, defined as light vs dark in the logistic model, was calculated for both colors to equal 0.929. The sensitivity was only highest for dark hair color (100%) when using the logistic model and was much lower for light hair color for the same reason (Table 2).

Predicting eye and hair color type separately using a multinomial logistic model yielded slightly higher accuracy compared to the Bayesian model. In any event, for both models the lowest AUC values were observed for intermediate eye color and for dark blond/light brown hair color. There is a possibility that the problem lies in imprecise color classification, which reflects uncertainties in distinguishing within intermediate eye color (green-eyed individuals and those with more pigments within the iris) and within different shades of hair color, which may be influenced by age-dependent hair color change during the entire lifetime of each individual (distinguishing between the dark blond and blond on one hand, and between light brown and brown on the other).

## Discussion

Eye and hair color represent human traits that have a potential to be predicted from genetic material with high reliability and used in expansive forensic investigations. In the present study, based on a new single multiplex of twelve SNPs, we confirmed the effect of five statistically most significant SNPs for eye and hair color in both statistical models. Specifically rs12913832 in *HERC2* showed the strongest association with both eye and hair color.

To illustrate the predictive performance of our single multiplex we focused on the five most statistically significant SNPs (rs1800407, rs7495174, rs12913832, rs1805008, and rs1393350). The other seven SNPs were not included, as being either monomorphic for our population or in linkage disequilibrium with other SNPs, or as just not being sufficiently statistically significant for prediction accuracy for Slovenian samples. For our study population, the only SNP being monomorphic was SNP rs1426654, as all volunteers were homozygote for the derived allele A/A. Our data were therefore inconclusive regarding the hypothesis of Velenzuela et al (19) that rs1426654 contributed to hair color variation. The single multiplex assay also included three SNPs (rs1129038 [4], rs7170989 [6], and rs1667394 [9]), which were previously recommended for eye and hair color prediction. For all these three SNPs, we concluded that they were in strong linkage disequilibrium with the other five most statistically informative SNPs, and it was therefore unlikely that their inclusion would have had any effect on the prediction power.

For genotyping accuracy of 105 Slovenian samples, this multiplex assay is for now designed to work optimally at 1 ng of template DNA, but its sensitivity has risen up to 62 pg by increasing the amount of AmpliTaq Gold DNA polymerase in reaction (5U). Our results on sensitivity and reliability of the multiplex could even be improved by increasing the injection and time voltage in capillary electrophoresis and also to use the polymer POP-7 instead of POP-4, due to its better mobility in the capillary of the ABI Prism 3130 Genetic Analyzer.

The single multiplex assay presented was a robust and sensitive DNA tool regarding amplification and regular determination of the homozygosity/heterozygosity for each SNP included. On the basis of these facts, the single multiplex assay could be suitable for the use in forensic casework due to its more efficient use of template DNA. Furthermore, even markers not significantly associated with a trait in a temporary study population can still independently contribute to the trait prediction in the enlarged Slovenian or even European population. After all, the growing number of known sequence variants that underlie the differences in human pigmentation may provide new SNPs with specific markers that we could include in our assay for a more accurate prediction of eye and hair color.
