# Supplementary material for: A single-nucleotide polymorphism (SNP) multiplex system: the association of five SNPs with human eye and hair color in the Slovenian population and comparison using a Bayesian network and logistic regression model
Source: Croat Med J. 2012 Oct;53(5):401–8. doi: 10.3325/cmj.2012.53.401 (PMC3490452; doi:10.3325/cmj.2012.53.401)
Supplement: Supplementary Table 1 [file CroatMedJ_53_s013.pdf]

Supplementary Table 1: SNP marker used in our assay for eye and hair color prediction with details on SBE primers.

| SNP-ID | gene | SBE primer length (bp) | SBE extension primer (5'-3') | Primer conc. (µM) | Alleles detected | Ref. |
|---|---|---|---|---|---|---|
| **rs1129038** | *HERC2* | 65 | GACTGACTGACTGACTGACTGACTGACTGACTGACTGACTTGCCTACAGTCTACAC AGCAGCGAG | 0,15 | A/G | Eiberg, 2008 |
| **rs12913832** | HERC2 | 25 | TGATGATAGCGTGCAGAACTTGACA | 1,50 | A/G | Eiberg, 2008 |
| **rs1393350** | TYR | 50 | GACTGACTGACTGACTGACTGACTGACTGAGTAAAAGACCACACAGATTT | 1,00 | A/G | self - designed |
| **rs7170989** | OCA2 | 60 | GACTGACTGACTGACTGACTGACTGACTGACGGGACTGCTGCTCCGCGATCT CACC | 0,25 | C/T | Duffy, 2007 |
| **rs7495174** | OCA2 | 40 | GACTGACTGACTGACTACGTCGCACCCGTCTGTGCACACT | 1,00 | A/G | Duffy, 2007 |
| **rs1800407** | OCA2 | 55 | GACTGACTGACTGACTGACTGACTGACTGACGTGGCCAGGCATACCGGCTCTCCC | 0,01 | A/G | Duffy, 2007 |
| **rs1667394** | OCA2 | 30 | GACTGAACGCAGCAATTCAAAACGTGCATA | 1,70 | A/G | self-designed |
| **rs26722** | *SLC45A2* | 40 | AATGCCAGCTCTGGATTTACGTAACCATTTTTAACTTTCT | 0,15 | C/T | Soejiam, 2007 |
| **rs16891982** | *SLC45A2* | 45 | GACTGACTGACTGACTAGAGGAGTCGAGGTTGGATGTTGGGGCTT | 0,65 | C/G | Soejima, 2007 |
| **rs1426654** | *SLC24A5* | 30 | ACCCTTGGATTGTCTCAGGATGTTGCAGGC | 0,05 | A/G | Soejima, 2007 |
| **rs1805005** | MC1R | 35 | GACTGACTGACTGACTGGTGGAGAACGCGCTGGTG | 0,15 | T/G | Mengel-From, 2008 |
| **rs1805008** | MC1R | 60 | GACTGACTGACTGACTGACTGACTGACTGACTGACTGAAGCATCGTGACCCT GCCG | 0,10 | T/C | Mengel-From, 2008 |
