# Supplementary material for: A single-nucleotide polymorphism (SNP) multiplex system: the association of five SNPs with human eye and hair color in the Slovenian population and comparison using a Bayesian network and logistic regression model
Source: Croat Med J. 2012 Oct;53(5):401–8. doi: 10.3325/cmj.2012.53.401 (PMC3490452; doi:10.3325/cmj.2012.53.401)
Supplement: Supplementary Table 2 [file CroatMedJ_53_s014.pdf]

Supplementary Table 2: SNP marker used in our assay for eye and hair color prediction with details on PCR primers.

SNP-ID	gene	forward primer	reversed primer	ref.
**rs1129038**	*HERC2*	CAGCAGCGACGATTCAGATA	ATCACGGCCAGTCAGTCTCT	Eiberg, 2008
**rs12913832**	HERC2	TGATAGCGTGCAGAACTTGACA	TAATTCAAAATGCCCCCAAG	Eiberg, 2008
**rs1393350**	TYR	GGTGAATGATAACACGAACAGATT	GATGCGTGCATATCCACCAACT	Sulem, 2007
**rs7170989**	OCA2	TCCCAATTTTTGTCCTCCTG	TTTTCCAGCCAACAAATGAA	Duffy, 2007
**rs7495174**	OCA2	TGCTGTCTGAACCTTTCCAA	GCTTAGGAAGCAAGGCAAGTT	Duffy, 2007
**rs1800407**	OCA2	TGAAAGGCTGCCTCTGTTCT	TATGGTCACAGGCGTGAAGA	Duffy, 2007
**rs1667394**	OCA2	TCCACCATTAAGACGCAGCAATTCAA	GAGAACTTTGAGGTCTCCAAC	Sulem, 2007
**rs26722**	*SLC45A2*	TGCCAGCTCTGGATTTACG	GCTGGGAAGACTGTTGGGTA	Soejiam, 2007
**rs16891982**	*SLC45A2*	GGAAAACACGGAGTTGATGC	CCAGAGGTGGAGAAGCAGAG	Soejima,2007
**rs1426654**	*SLC24A5*	TTCAGCCCTTGGATTGTCTC	AATTGCAGATCCAAGGATGG	Soejima, 2007
**rs1805005**	MC1R	CTGGTGAGCTTGGTGGAGA	TCCAGCAGGAGGATGACG	Mengel-From, 2008
**rs1805008**	MC1R	CTGTCCAGCCTCTGCTTCCT	GGCCACGTGGTCGTAGTAGG	Mengel-From, 2008
