# Supplementary material for: A single-nucleotide polymorphism (SNP) multiplex system: the association of five SNPs with human eye and hair color in the Slovenian population and comparison using a Bayesian network and logistic regression model
Source: Croat Med J. 2012 Oct;53(5):401–8. doi: 10.3325/cmj.2012.53.401 (PMC3490452; doi:10.3325/cmj.2012.53.401)
Supplement: Supplementary Table 3 [file CroatMedJ_53_s015.pdf]

Supplementary Table 3: Allele frequency for the selected twelve SNPs in Slovene population compared with data from the International HapMap consortium (CEU).

SNP locus	allele	105 Slovene samples	HapMap (CEU)
rs1129038	allele G	0,338	/
	allele A	0,662	/
rs12913832	allele G	0,662	0,792
	allele A	0,338	0,208
rs1393350	allele G	0,780	0,774
	allele A	0,220	0,226
rs1426654	allele A	1,000	1,0
	allele G	0,000	/
rs1667394	allele C	0,250	0,144
	allele T	0,750	0,856
rs16891982	allele C	0,033	0,016
	allele G	0,966	0,984
rs1800407	allele T	0,119	0,075
	allele C	0,881	0,925
rs1805005	allele G	0,950	0,920
	allele T	0,050	0,08
rs1805008	allele C	0,880	0,872
	allele T	0,120	0,128
rs26722	allele C	0,940	0,996
	allele T	0,060	0,004
rs7170989	allele C	0,630	0,804
	allele T	0,370	0,196
rs7495174	allele A	0,890	0,958
	allele G	0,110	0,049
